# The threaten of typhoons to the health of residents in inland areas: a study on the vulnerability of residents to death risk during typhoon “Lekima”: In Jinan, China

**DOI:** 10.1186/s12889-024-17667-y

**Published:** 2024-02-26

**Authors:** Yiwen Ma, Xianhui Zhang, Yingjian Zhang, Jipei Du, Nan Chu, Jinli Wei, Liangliang Cui, Chengchao Zhou

**Affiliations:** 1 School of Public Health, Cheeloo College of Medicine, Shandong University, 44 Wen-hua-xi Road, Jinan, Shandong 250012 China; 2 Jinan Municipal Center for Disease Control and Prevention, affiliated to Shandong University, 2 Weiliu Road, Huaiyin District, Jinan, 250021 China; 3 NHC Key Lab of Health Economics and Policy Research, Shandong University, 44 Wen-hua-xi Road, Jinan, Shandong 250012 China; 4 Institute of Health and Elderly Care, Shandong University, 44 Wen-hua-xi Road, Jinan, Shandong 250012 China

**Keywords:** Inland typhoon, Mortality, Vulnerability, Time-stratified case-crossover

## Abstract

**Background:**

Studies had suggested increased risk of death of residents was associated with typhoons, particularly coastal regions. However, these findings ignored the impact of inland typhoons on the health of residents, especially the indirect death risk caused by typhoons. This study aimed to investigate the acute death risk of residents during inland typhoon Lekima in Jinan, further identify vulnerable populations and areas.

**Methods:**

We selected the daily death from 11 to 27th August 2019 in Jinan as case period, and conducted a time-stratified case-crossover design to match the contemporaneous data from 2016 to 2018 as control period. We used the generalized linear Poisson models to estimate the related effects of death risk during typhoon Lekima and lag days.

**Results:**

During the Lekima typhoon month, there were 3,366 deaths occurred in Jinan. Compared to unexposed periods, the acute death risk of non-accidental diseases (especially circulatory diseases), female and the older adults increased significantly in the second week after the typhoon. The maximum significant effect of circulatory disease deaths, female and older adult deaths were appeared on lag9, lag9, and lag13 respectively. And the typhoon-associated RR were 1.19 (95%CI:1.05,1.34), 1.28 (95%CI:1.08,1.52), and 1.22 (95%CI:1.06,1.42) respectively. The acute death risk of residents living in TQ and CQ increased significantly on Lag2 and Lag6 after the typhoon, respectively, while those living in LX, LC, HY, JY, and SH occurred from Lag 8 to Lag 13 after the typhoon. LC lasted the longest days.

**Conclusions:**

Typhoons would increase the vulnerability of residents living in Jinan which mainly occurred from the seventh day after the typhoon. Residents suffering from non-accidental diseases (circulatory diseases), female and the older adults were more vulnerable. The vulnerability of TQ and CQ occurred on Lag2 and Lag6 after typhoon Lekima, respectively, and the other areas except ZQ and PY occurred from Lag 8 to Lag 13. LC lasted the longest duration. Our findings emphasized the importance of the emergency response, which would help policymakers to identify vulnerable regions and populations accurately during typhoons and formulate the emergency response plan.

**Supplementary Information:**

The online version contains supplementary material available at 10.1186/s12889-024-17667-y.

## Introduction

Global warming aggravates the instability of the climate system, leading to the increasing frequency of extreme weather events [1, 2]. Typhoon disasters, as extreme weather phenomenon, have occurred in many countries in recent years. China is one of the most vulnerable countries to typhoon disasters currently [3–5]. There are seven typhoons or tropical cyclones that make landfall from coastal provinces of China each year, and the spatial patterns of the disasters for typhoons with different landing paths were significantly different [6, 7].

Typhoon is familiar with its impact on the coastline, such as storm surge, strong wind. Most typhoons weaken rapidly once they encounter land [8]. With the depletion of storm “fuel” –– the heat and water extracted from the ocean, the force of typhoon gradually weakens. However, with global climate change, the typhoon hazards have become unpredictable. Jeffrey confirmed that the lethal range of the typhoon was not limited to the coastline [9]. The “inland stage” of typhoon ––namely, the second storm, may cause more deaths and property losses in inland areas than those in coastal areas. The inland areas are located far from the coastline and major rivers [10]. At the initial stage of the typhoon's migration to inland areas, its power has weakened, but it may rejuvenate or re-strengthen after the transition of temperate climate. After the transition, the typhoon becomes a dangerous mixed cyclone. The impact of “second typhoon” on inland areas is diverse, and the common denominator is destruction [8]. The chain disasters caused by typhoons, such as rainstorm and flood, have produced hundreds of fatalities. This thus, the vulnerability of inland areas due to the second typhoons should be paid more attention to.

Typhoon Lekima, the most powerful typhoon in 2019, made landfall in Wenling City, Zhejiang Province, China on August 10. The maximum wind speed reached level 16. Typhoon Lekima affected 14,024,000 Chinese residents, caused 57 deaths, 14 missing, and 2,097,000 residents to require emergency care. The direct economic loss was more than 8.43 billion US dollars, and the indirect economic loss was unquantifiable, especially the health risk caused by typhoon disaster was enormous, which was difficult to estimate [2, 5, 11]. In August 11, the typhoon Lekima landed in Jinan, and had a devastating impact on Jinan in just three days. As the capital of Shandong Province, Jinan has become the core connection center of the Yangtze River Delta and Beijing-Tianjin-Hebei economic zones, with a population of nearly 7.6 million in 2019. It is a typical inland city in China [12]. Previous studies showed that compared with other inland cities, the vulnerability index of Jinan exposed to typhoon is over 0.2, which belongs to a highly vulnerable city [13, 14]. Therefore, it is representative to explore the typhoon Lekima and Jinan.

Many previous studies have illustrated that increased risk of death of residents was associated with typhoons. Existing studies mainly focused on the total death distribution or the incidence of infectious diseases of residents in the coastal areas [15]. The reported results only covered a short period, which paid more attention to the direct fatalities during the typhoon days [16, 17]. It is little known about the impact of typhoons on the health of inland residents, especially in China. However, many findings had shown that from 1970 to 1999, typhoon-related deaths mainly occurred in inland areas, especially in areas that had never experienced typhoons [13, 18, 19]. There were more vulnerable in residents who lack experience in resisting typhoon disasters (adaptive behavior) [15]. Additionally, the health impact caused by the “inland stage” of typhoons is not limited to the direct effect, and the threat brought by the lag effect cannot be ignored. McKinney found that the direct death caused by typhoons accounted for only 4%, and the indirect death caused by typhoon-related cardiovascular diseases, malignant tumors, diabetes and accidents accounted for 67% [20]. Therefore, it is vital to explore indirect death risk (lag effect) caused by typhoons in inland areas.

This study aims to identify the vulnerability of residents in Jinan on typhoon Lekima. Specifically, we will first investigate the acute effect of typhoon Lekima on mortality of residents in Jinan. Second, we will explore the difference of acute death risk among different subgroups during typhoon Lekima. This study will provide scientific evidence about emergency response in extreme weather in inland.

## Methods

### Study area

Jinan is located in the middle of Shandong Province. The geographic position is between 36◦01’N ~ 37◦32’N and 116◦11’E ~ 117◦44’E [12]. Now, Jinan City has jurisdiction over ten districts of Lixia, Shizhong, Huaiyin, Tianqiao, Licheng, Changqing, ZhangqiuJiyang, Laiwu and Gangcheng and two counties of Pingyin and Shanghe. Then, the mountainous area mentioned in this study is Nanshan, which belongs to a part of Licheng District, Jinan City. Since Gangcheng and Laiwu were divided into Jinan in 2019, we excluded the two districts in the data screening [21].

### Data collection

The daily mortality data were obtained from the report of Jinan Municipal Center for Disease Control and Prevention from 2016 to 2019. Data used in this study included the time of death, the cause of death, the demographic characteristics of the deceased individual. Cause of death in this study was coded according to the International Classification of Disease, Revision 10 (ICD-10, 2012 version). Six causes had been aggregated, including all-cause disease, accidental disease (ICD-10: S00–Z99), cancer (C00-D48), non-accidental disease (A00-R99), circulatory disease (I00–I99), and respiratory disease (J00–J99). Additionally, we had excluded the non-Jinan residents based on the residential address. We have consulted the demographic data from the Jinan Statistical Yearbook-2020, and we found that the population of natural growth rate decreased year by year from 2016 to 2019 [21]. Therefore, the population of growth can not affect our results.

Daily weather data for the same period were collected from the China Meteorological Science Data Sharing Service Network. Daily average hourly air pollutant data were collected from Environmental Monitoring Center of Jinan from 2016 to 2019. The data included daily average temperature (T average), daily maximum (T max) and minimum temperature (T min), daily average relative humidity (RH), daily average rainfall, daily average air pressure, daily average wind speeds, CO, SO_2_, NO_2_, O_3_, and PM_10_. Previous studies showed that exposure to a certain level of air pollution might increase the acute onset of diseases, which would lead to death. Therefore, we added pollutants to test the stability of the main model [22].

### Statistical Analysis—Time-stratified case-crossover study

We used the time-stratified case-crossover method to explore the acute effect of the Lekima typhoon on health, which had been verified as an efficient approach to result in unbiased conditional logistic regression estimates [23]. The basic principle of time-stratified case-crossover design is to stratify, and the case phase and control phase are at the same time layer [24]. Several control periods are randomly distributed, and the case period is not fixed in a certain position. The distribution of these control periods may be located before the case period, either after the case period, or scattered before and after the case period [25, 26]. Existing research found that it could lead to biased estimates when studying disasters if disasters have extended impacts on health risks and if this period of potential extended disaster effects is incorrectly specified in the model [27, 28]. Given these potential concerns with applying the time series or case-crossover study designs to analyze the effects of a disaster, we used a study design that matched the days of the typhoon Lekima to unexposed days from the same time of the year in other years in Jinan, with control for long-term trends in mortality rates and the influence of temperature on mortality risk incorporated in the statistical model fit to this matched data. Meanwhile, previous studies have used similar study design to investigate hospitalization risk of other natural disasters, including heat waves, wildfires and floods [27, 29, 30]. We had used to plan to select the same period of 2016–2018 and 2020 as the control periods. Since the 2020 was the epidemic year of COVID-19, the population mobility pattern, health outcomes (deaths), social and economic development had undergone significant changes, we had to exclude 2020.

Firstly, we conducted a Spearman analysis on the correlation between the meteorological variables and pollutant data, and referred to the results of similar studies. We determined the confounding factors contained in the main model, including the daily maximum temperature (T max). According to the principle of strict control selection, we also considered for long-term trends, the effects of the day of the week, the dates of the same month, and the same week. Then, based on the results of the preliminary exploration analysis, we determined that the lag effect of typhoon lasted for 14 days (Lag1-14). Finally, we fit the following generalized linear model, using the quasi-poisson distribution to explore the acute effect of the Lekima typhoon. The main model was as follows:

$$\mathrm{Log}\left(\mathrm E\left[{\mathrm Y}_{\mathrm t}\right]\right)=\mathrm\alpha\;+\upbeta\mathrm{X}\;+\;\mathrm{Tmax}\;+\mathrm{stratum}$$

t was the observational day/observational period;

Y_t_ was the number of all-cause deaths on the observational day;

α was the intercept term;

β was vector of coefficients;

X was the classified variable of “1” and “0” in typhoon event. The period affected by typhoon Lekima was defined a typhoon event and simulated the typhoon event with binary variables. The typhoon event was recorded as 1, and other day was recorded as 0. The contemporaneous data from 2016 to 2018 of days were chosen as a control;

stratum was the categorical variable, which was the matching variable of month and week.

### Sensitive analysis

We carried out the sensitivity analysis to assess the stability of the model results. First, by adjusting PM_10_, O_3_, NO_2_, SO_2_, CO respectively, we controlled the potential interference factors–-atmospheric pollutants, which may affect the association between typhoon and mortality. The degree of change of the impact of typhoon on mortality was further observed. Next, the effects of daily average temperature, daily average relative humidity, daily average air pressure, daily minimum temperature, and daily average wind speed were controlled.

All analyses and regression model analyses were performed using R 4.1.2 and casecross from the “season” package [31]. We used the relative risk (RR) and 95% confidence interval (CI) as the estimated expression of the death effect in typhoon in the regression model. The risk ratio estimates generated from case-crossover data were directly comparable to those generated by other considered study designs.

## Results

### Descriptive analysis results

Table 1 shows the distribution of daily deaths, meteorological and air pollutant variables in Jinan during August 2019.

**Table 1:** Distribution of daily deaths and meteorology conditions in August 2019, Jinan City

Variable	*N* (%)	Mean (SD)	Min	Max
Daily death counts				
All-cause	3366.0	108.6(11.1)	78.0	130.0
Accidental	191.0(5.7)	6.2(2.8)	1.0	13.0
Non-accidental	3175.0(94.3)	102.4(10.5)	74.0	124.0
Circulatory	1674.0(49.7)	54.0(7.8)	33.0	68.0
Respiratory	253.0(7.5)	8.2(2.9)	4.0	14.0
Cancer	978.0(29.1)	31.6(4.5)	22.0	40.0
Gender				
male	1915.0(56.9)	61.8(6.5)	45.0	72.0
female	1451.0(43.1)	46.8(7.4)	33.0	64.0
Age				
Old (≥ 65)	2508.0(74.5)	80.9(9.5)	51.0	98.0
65–74	853.0(25.3)	27.5(5.3)	16.0	39.0
75–84	956.0(28.4)	30.8(6.6)	21.0	44.0
≥ 85	699.0(20.8)	22.6(5.6)	10.0	33.0
Young (< 65)	858.0(25.5)	27.6(5.9)	19.0	43.0
Residence				
Lixia (LX)	323.0(9.6)	10.4(3.2)	3.0	19.0
Licheng (LC)	531.0(15.8)	17.1(3.9)	8.0	24.0
Shizhong (SZ)	352.0(10.5)	11.4(3.6)	3.0	17.0
Huaiyin (HY)	214.0(6.4)	6.9(2.9)	2.0	12.0
Tianqiao (YQ)	284.0(8.4)	9.0 (3.2)	2.0	15.0
Changqing (CQ)	324.0(9.6)	10.5(3.9)	4.0	21.0
Pingyin (PY)	155.0(4.6)	5.0(2.2)	1.0	10.0
Jiyang (JY)	288.0(8.6)	9.3(4.2)	2.0	22.0
Zhangqiu (ZQ)	575.0(17.1)	18.6 (3.9)	10.0	28.0
Shanghe (SH)	316.0(9.4)	10.2 (3.2)	3.0	18.0
Meteorological				
T.max (°C)	31	30.1(3.3)	22.5	35.6
T.average (°C)	31	25.6(2.2)	21.0	29.8
T.min (°C)	31	22.1(2.3)	18.2	26.4
Relativehumidity(RH,%)	31	69.7 (15.5)	40.0	98.0
Rainfall(m)	31	11.8 (32.5)	0	148.9
Wind(m/s)	31	1.9 (0.7)	1.1	4.0
Pressure(kPa)	31	987.2 (4.8)	975.1	994.6
Air pollution				
PM_10_ (μg/m3)	31	57.0(27.0)	5.0	117.0
O38h((μg/m3))	31	135.0(43.0)	62.0	215.0
SO2 (μg/m3)	31	9.0 (2.0)	6.0	14.0
CO((μg/m3))	31	796.0(244.0)	418.0	1384.0
NO_2_ (μg/m3)	31	30.0 (11.0)	9.0	55.0

In this study, 3,366 deaths occurred in the Lekima typhoon month, and the daily deaths was 108.6. Among them, there were 1,915 male residents died, accounting for 56.9%, and 43.1% were female residents. The proportion of deaths among the older residents (≥ 65) was significantly higher than that of other age subgroups, accounting for 74.5% of all deaths. We analyzed the proportion of deaths in ten districts of Jinan respectively. The results indicated that ZQ had the highest proportion of death, followed by LC, accounting for 17.1% and 15.8% of the total death respectively. For the Lekima month, the all-cause mortality was 44.4 per 100 000 person-years, 3,175 deaths occurred in non-accidental cases, accounting for 94.3% of all deaths, and accidental cases accounted for 5.7%. An average of 102.4 deaths of non-accidental occurred per day, including 54.0 deaths from cardiovascular diseases, 31.5 from cancer, and 8.2 from respiratory diseases in August 2019.

During typhoon Lekima (from August 11 to August 13), the daily maximum temperature, daily average temperature and daily minimum temperature decreased to the lowest value, while the wind speed and rainfall reached the extreme value of this month, and the air pressure decreased slightly. The value of air pollution factors decreased significantly.

### The effect during Typhoon Lekima

**Fig. 1:**
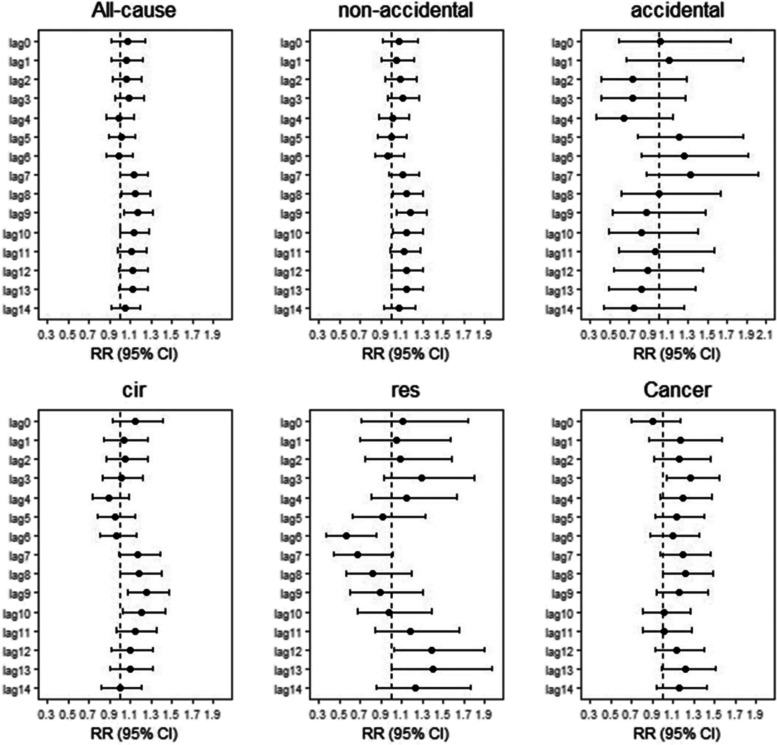
Summary single-day lag risk (RR, 95% CI) of death on Jinan residents with different disease during Typhoon. *Lag 0: the relative risk of mortality for different diseases on the typhoon day; Black dots show estimates; horizontal lines show 95% confidence intervals. The black horizontal line shows as a reference a relative risk of 1

As can be seen in Additional File 1, compared to matched non-typhoon reference period from 2016 to 2018, just the proportion of accidental disease deaths among residents in Jinan increased slightly during the typhoon month (August, 2019), and there was no increase in all cause and non-accidental deaths. The risk of all-cause deaths increased by 7.12% on Lekima typhoon days (95% CI: 0.92–1.24), but there was no statistical significance. As can be seen in Fig. 1**,** we found that the risk of all-cause death began to increase on Lag7 after the typhoon, and the non-accidental deaths risk was on Lag8. The maximum single-day effect was all observed at Lag9, with a RR of 1.17(95%CI:1.04–1.31) and 1.19 (95%CI:1.05–1.34) respectively, then diminished gradually. For non-accidental disease, a significant increase for the risk of circulatory deaths was occurred from lag8 to lag10 and reached a maximum effect at lag9 (RR = 1.26, 95% CI:1.07–1.47). The risk of respiratory deaths increased significantly only at lag12 and lag13, and the maximum effect was observed at lag13 (RR = 1.41, 95%CI:1.00–1.97). The risk of cancer deaths was significant at lag3 with a RR of 1.27 (95% CI: 1.03–1.55).

**Fig. 2:**
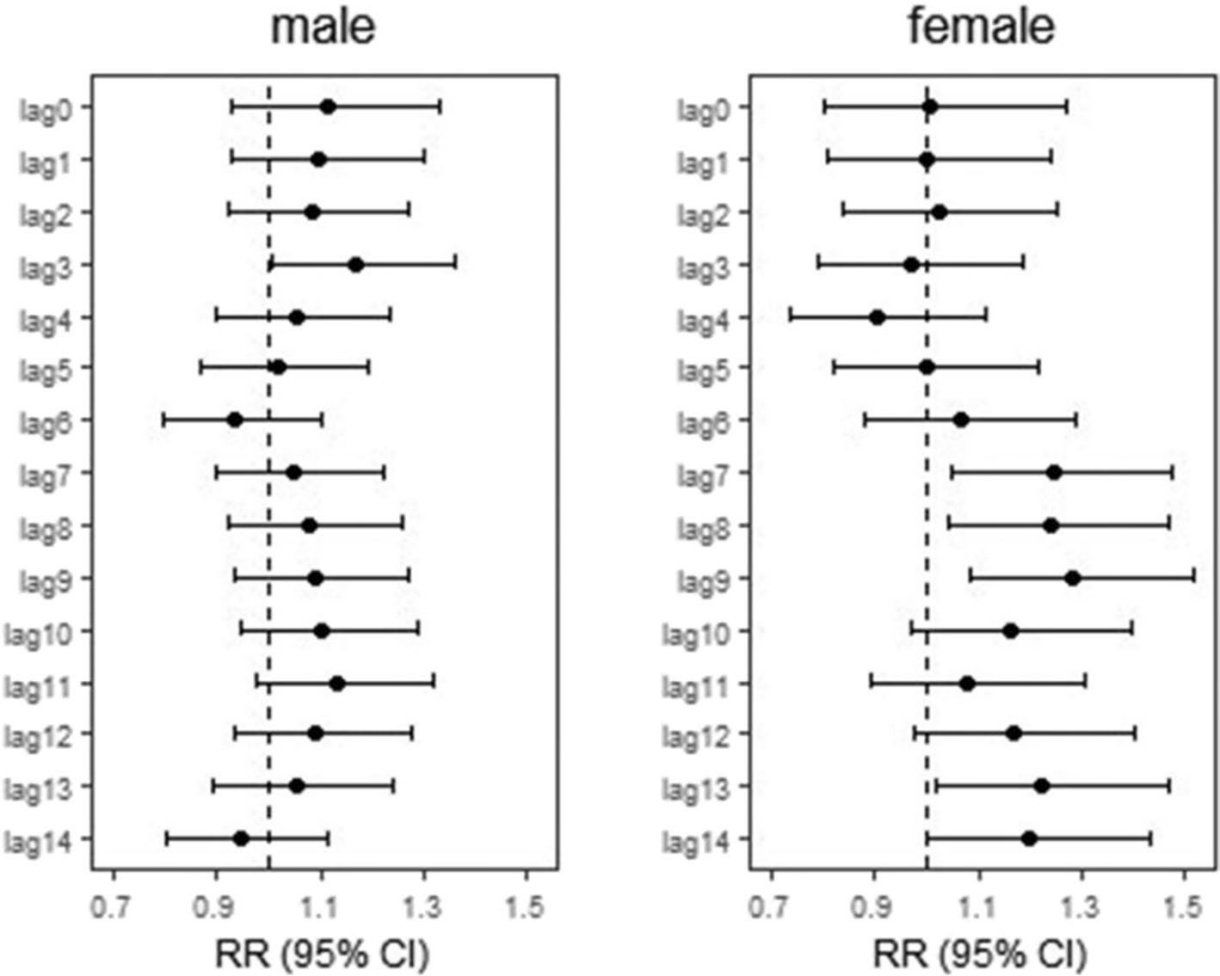
Summary single-day lag odd risk (RR, 95% CI) of death on Jinan residents with different gender during Typhoon

**Fig. 3:**
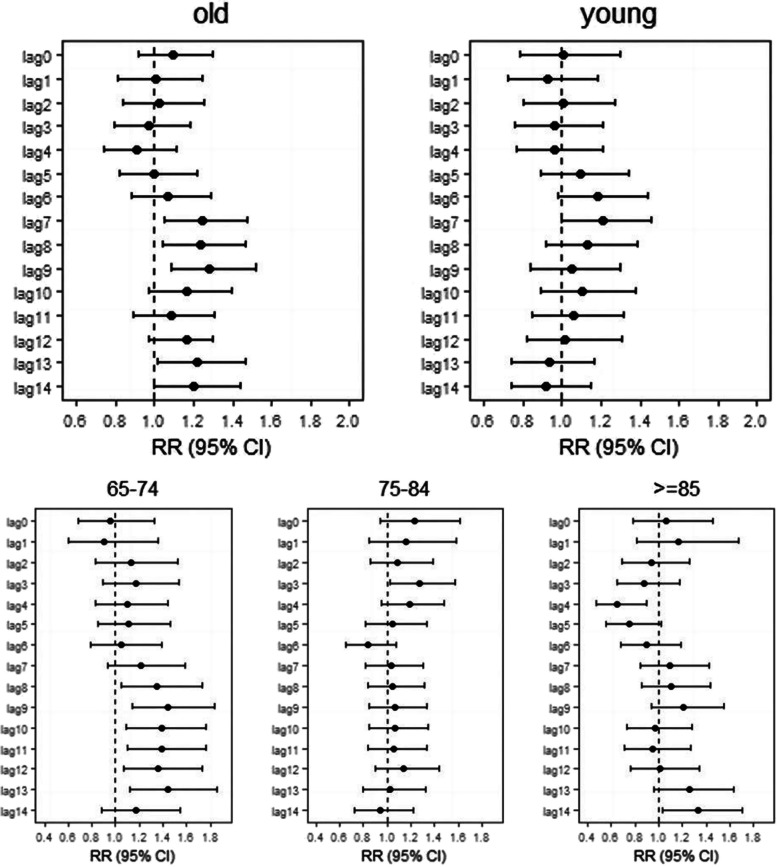
Summary single-day lag odd risk (RR, 95% CI) of death on Jinan residents with different age groups during Typhoon

Stratified analysis results illustrated the death risk of Jinan residents between different gender, age and residence in the Lekima typhoon event. Additional File 2 and Additional File 3 shows that there was no significant increase in the number of deaths among different genders and age groups during the typhoon. As can be seen in Fig. 2, the significant increase in risk of death among the female occurred on the seventh day after the Lekima typhoon, lasted for three days, and reached the maximum on lag9 (RR = 1.28,95%CI:1.08–1.52). For the men, the risk of death increased significantly only at lag3, with a RR of 1.67 (95% CI: 1.09–1.32). In Fig. 3, we found that the significant increase in the death risk of the older adults (≥ 65), especially in 65–74 age group, was observed on the second week after typhoon (lag9,12,13) and with a maximum at lag13 (RR = 1.22, 95% CI:1.06–1.42). There was no evidence the typhoon could increase the risk of deaths among young people (< 65).

**Fig. 4:**
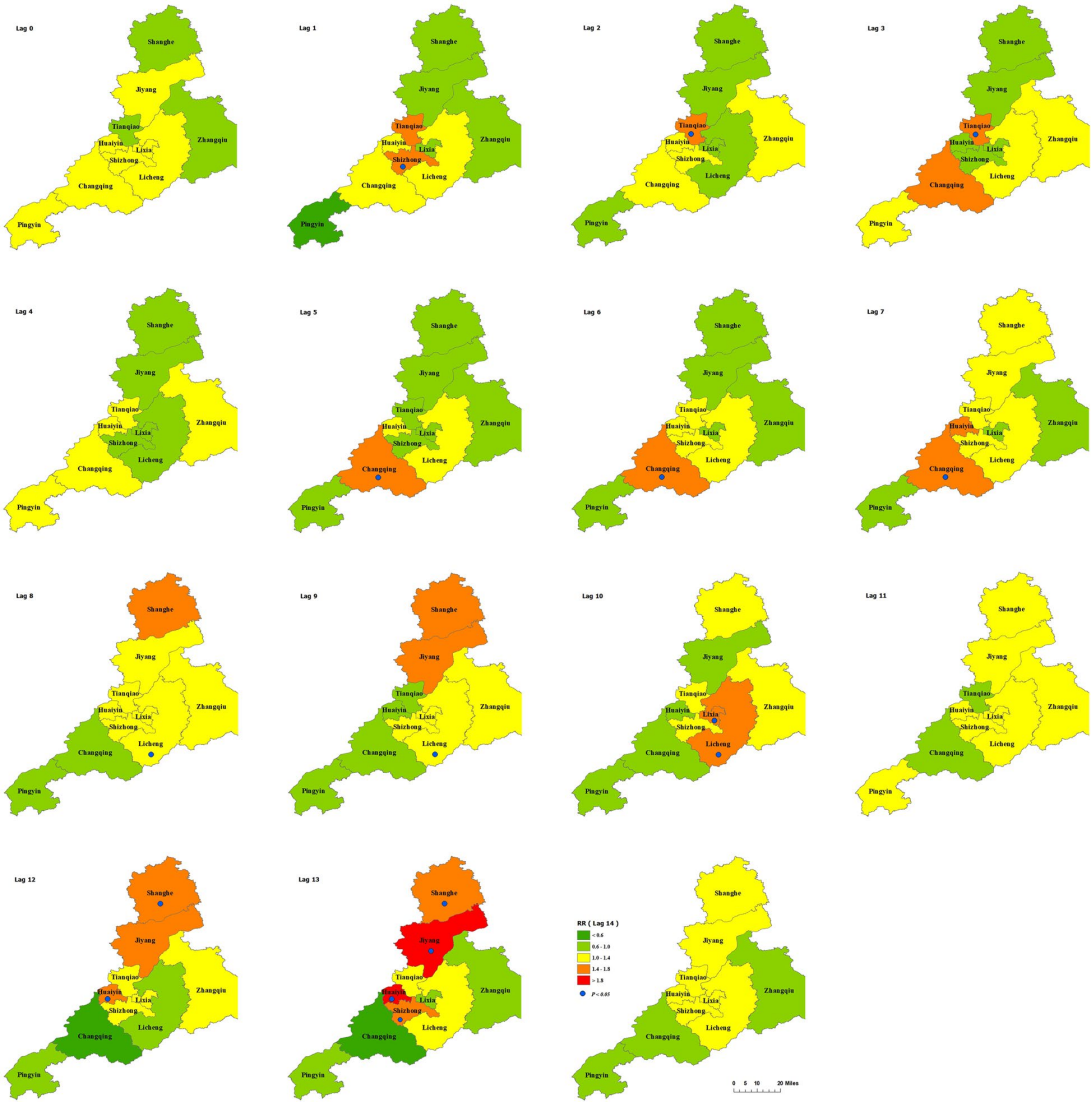
Summary single-day lag odd risk (RR, 95% CI) of death on Jinan residents with different districts during Typhoon. *RR ≥ 1,Yellow and above pixels; the blue circular: the areas with significant risk of death

As can be seen in Additional File 4 and Fig. 4, although there was a slight increase in the number of deaths during the typhoon, which occurred in LX, LC, SZ, HY, and TQ, the increased risk of mortality was not significant. The acute death risk of residents living in TQ and CQ increased significantly on Lag2 and Lag6 after the typhoon, respectively, while those living in LX, LC, HY, JY, and SH appeared from Lag 8 to Lag 13 after the typhoon. The acute mortality risk of residents in LX and LC reached the maximum on Lag10, with a RR of 1.44(95%CI:1.02–2.03) and 1.45 (95%CI:1.09–1.93) respectively, and with no significant risk after Lag10. The acute death risk in LC lasted the longest days (three days). The acute mortality risk for residents who lived in SH appeared on Lag12, and the residents who SZ, HY, and JY appeared on Lag13. There was no evidence that the typhoon was associated with the death risk of resident in ZQ and PY.

### Sensitivity analysis

**Fig. 5:**
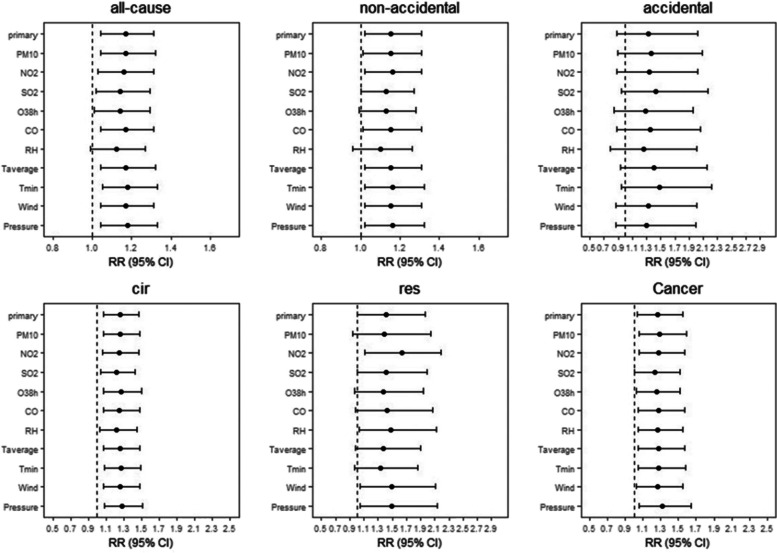
The effects of typhoon on the death of different diseases for Jinan residents when changing the temperature, meteorological factor and pollutant

**Fig. 6:**
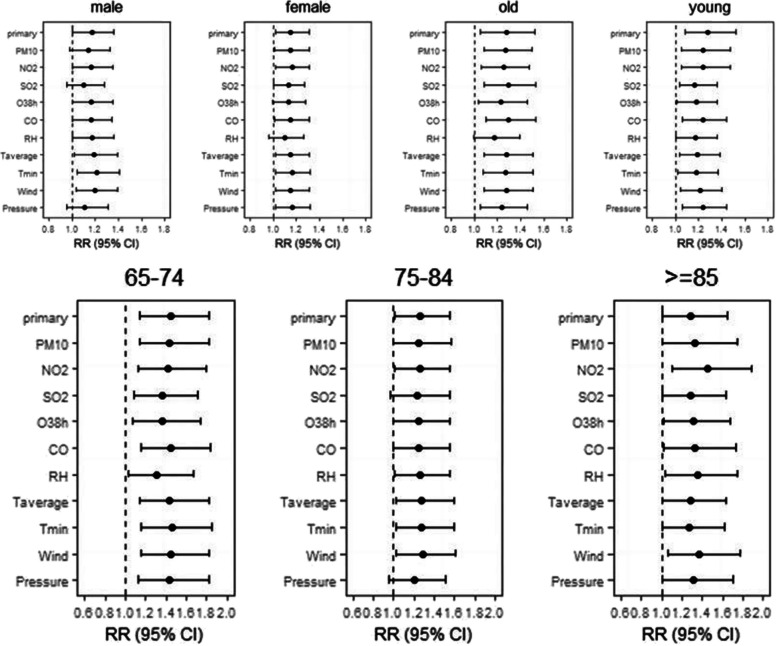
The effects of typhoon on the death of different gender and age groups for Jinan residents when changing the temperature, meteorological factor and pollutant

**Fig. 7:**
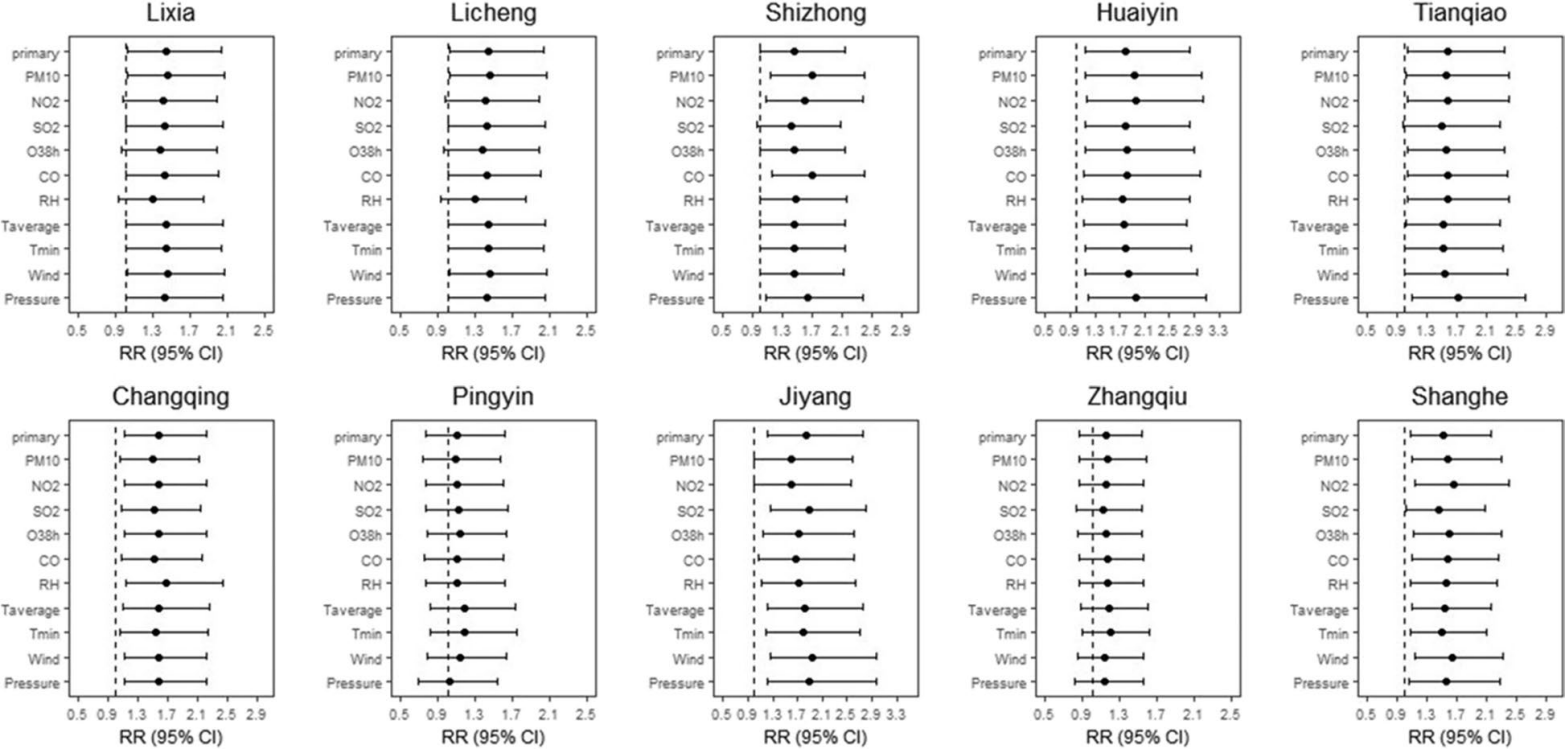
The effects of typhoon on the death of different districts for Jinan residents when changing the temperature, meteorological factor and pollutant

The sensitivity analysis results in Fig. 5, Fig. 6, and Fig. 7. The results showed the model changed only minimally after controlling for the air pollutants and the meteorological factors, which suggested that the main model was of good fit and the result was stable. Estimates of RRs were robust to modeling choice and the matching method for selecting unexposed days, when adding the daily max temperature in the main model.

## Discussion

This study focused on the health vulnerability of vulnerable groups affected by climate change. We conducted a comprehensive exploration of the concept of health vulnerability. In the research about health vulnerability, we regarded typhoon as a disaster-causing factors, and comprehensively analyzed the acute death risk of residents during Lekima for the purpose of reducing population health vulnerability. Our research not only enriched the findings of environmental health, but also could provide a new theoretical perspective.

Our findings showed that the acute death risk of residents living in Jinan increased significantly in two weeks after the typhoon Lekima. The vulnerability of residents with different disease types, gender, age, and regions was different. The risk of direct death among residents in Jinan did not increase during Lekima, which was consistent with a previous study in Guangzhou [32]. The possible reason was that typhoon warning system has been continuously improved. The advanced warning system would help the public capture the forecast and real-time information of typhoon easily, which played an important role in emergency evacuation. Additionally, previous studies showed that the shocking adaptability among residents to typhoons might reduce of direct mortality [9].

Our study found that the increased risk of death from non-accidental diseases was significant from the Lag8 after the typhoon, with cardiovascular disease occurring earliest and lasting the longest. There was no evidence that the typhoon was associated with an increased risk of accidental death. Compared with accidental death (such as drowning death), it was difficult to capture the increased risk of circulatory with traditional disaster monitoring methods [27]. These non-accidental deaths were not directly caused by the physical forces of typhoons, nor were they rare during non-typhoons [14, 27]. During the landing of Lekima, the rainstorm caused by the typhoon flooded many roads, destroyed lots of trees and buildings, and interrupted public transport in Jinan. The possible reason for the increased risk of cardiovascular disease death was related to the disruption in treatment of chronic conditions caused by blocked transportation and damaged communication infrastructure [33]. It will take some time to clean up the damaged roads and restore communications, which might cause residents with circulatory diseases to miss the prime treatment time. Traumatic psychological consequences and a high prevalence of anxiety-mood disorders were evident after Typhoon, that might also a significant reason for the increase in heart attacks and cardiac arrest [16, 34]. Thus, the typhoon could be likened to an emotional button, which would exacerbate the risk of acute myocardial infarction [27]. In addition, the typhoon made landfall during the high temperature period, the combined effect of high temperature and humidity might lead to an increase in blood viscosity and cardiac output which may result in dehydration, hypotension, surface blood circulation increases and even the impairment of peripheral vascular endothelial function, then contribute to mortality events [35–37]. We also had found a slight correlation between the risk of respiratory death and this typhoon. The increased risk of death from respiratory diseases was significant from the Lag12 after the typhoon and lasted for two days. A few previous epidemiological studies enlightened us that the high morbidity of chronic obstructive pulmonary disease during typhoons would increase the death risk of respiratory diseases [38, 39]. The deaths of respiratory disease might be related to disrupted power supplies for breathing aids. Meanwhile, the high winds could spread the particulates into the air, exacerbating chronic respiratory diseases [40].

Interestingly, we found that the significant increase in risk of death among female occurred on Lag7 after the storm and lasted for three days. This phenomenon was not found in the male group, which was contrary to the results of a Guangzhou study [32]. Compared with Guangzhou, the traditional Chinese family value of female residents living in Shandong were deeply ingrained. However, family value emphasized traditional sense of responsibility and belongingness. Female residents had suffered most of the housework burden at home and dedicated a lot to their family. The chain disasters caused by typhoon greatly increased their physical and psychological burden. Second, women, as a relatively vulnerable group, were more difficult to obtain the economic opportunities and productive resource opportunities to deal with climate change [41]. Then, a few previous epidemiological studies enlightened us that female residents suffered from greater psychological pressure than men after natural disasters, and were more likely to show their vulnerability to psychological and physical health [42, 43].

Consistently, the older adults (≥ 65) were more vulnerable than the young group during the typhoon. Bell found the sizeable increase in older inpatients during eight separate hurricanes of American [44]. Weinberder referred that the increase in the proportion of emergency department visits due to cardiovascular disease, respiratory disease and kidney disease among the older adults (≥ 65) was associated with Hurricane Sandy [14]. These results added further evidence to prove that typhoons would increase the risk of death and illness of the older adults through multiple pathways. Several possible explanations for this finding are as follows. First, with the gradual aging and the decline of physical function among older adults (such as mobility inconvenience), the adaptability to the changes of the external environment might be worsen. Especially, when expose to high temperatures, elderly persons’ ability to regulate their body temperature is weaker than that of the young [45, 46]. Second, the high prevalence of chronic diseases in older adults would increase their psychosocial stress. The risk of death in older adults increased due to the lack of routine medical care and drugs when the typhoon landed [27, 33, 47, 48]. Additionally, we also explored finer strata of age which showed that the typhoon had an impact on the increased risk of death among 65–74 age group.

Our study showed that the acute death risk of Jinan residents living in different areas appeared at different point-in-time during typhoon Lekima. The acute death risk of residents in TQ and CQ appeared earlier, and those living in other areas (except ZQ and PY) appeared from Lag8 to Lag13 after the typhoon. LC and ZQ as the typhoon landing areas, we found that the risk of acute death in LC increased during the typhoon, while ZQ did not. The reason for the phenomenon might be related to the timely and sufficient emergency rescue operations during this typhoon. ZQ was located in the plain, which allowed rescuers to carry out emergency rescue operations in time and evacuate affected residents. Meanwhile, the government agencies prepared the emergency shelters, which equipped with clean drinking water, sufficient daily supplies and comprehensive medical services [49]. The comfortable environment might reduce the spread of infectious diseases after disasters, pacify the panic of victims, improve the quality of life, then reduce the lag effect on health caused by the typhoon [50]. Although LC also carried out rescue operations timely, the geological features of vertical and horizontal gullies in Nanshan of LC made the rescue mission difficult [51]. In addition, the severe destruction to local infrastructures caused by this typhoon might also increase the risk of direct or indirect death among residents. Therefore, when formulating the emergency planning for typhoon, it was necessary to pay more attention to the mountainous terrain along the track of typhoon [6].

In conclusion, we provide some specific recommendations. Firstly, the government agencies should improve their emergency and disaster management response mechanisms to reduce the threat to life caused by subsequent typhoons. Secondly, the community agencies should improve the ability of dealing with emergency, such as conducting knowledge seminars on disasters, carrying out evacuation drills, while guiding residents to cultivate their crisis awareness. Meanwhile, when receiving a typhoon warning signal, community agencies should inform residents with chronic diseases to prepare sufficient treatment drugs and equipment in advance. Thirdly, rescuers need to carry out emergency rescue operations in time and evacuate affected residents during the typhoon landing period. As for areas where are hard to carry out rescue missions, the government agencies should organize residents to evacuate in advance and prepare temporary accommodation for them. Then, the temporary accommodation should equip with clean drinking water, sufficient daily supplies and comprehensive medical services. Finally, the psychological issues of victims cannot be ignored. Professionals should conduct psychological counseling of anxiety and depression regularly, especially for women and the older adults.

Our study had several strengths. To our knowledge, it was the first Chinese study to discuss the vulnerability of inland area during typhoons. Second, the accurate and reliable datum ensured the credibility of this research. Third, compared with the traditional design, we used the time-stratified case-crossover design to control the temporal trend automatically. Then, we compared the death in the unexposed period from 2016 to 2018 with the five-day moving average death of residents in August 2019, which made our results more credible.

### Limitations

There were several limitations in our research. First, we had less information and about why the mortality risk of inland residents did not significantly increase during the first week, and the specific pathogenesis of some diseases. Second, this study did not consider the impact of economic factors, humanistic factors, medical and health service on the mortality of residents, which warranted further detailed investigation. Finally, due to the limitations of existing data, our discussion just focused on the overall reasons for the significant risk of acute death, rather than on the single date exceedance, and our results might not be generalizable to other inland areas.

## Conclusion

This study demonstrated an acute death risk of residents during inland Typhoon with individual and regional scale characteristics as subgroup to identify the vulnerability during typhoons. The typhoon Lekima had increased the vulnerability of Jinan residents, and the mortality risk of different vulnerable groups appeared at different point-in-time. From the seventh day after Typhoon Lekima, the risk of death among residents with circulatory system diseases, women, the older adults increased significantly. The significant risk of death for most vulnerable groups appeared on Lag8, reached the maximum on Lag9 and Lag10. And the risk among residents living in TQ and CQ increased significantly on Lag2 and Lag6, respectively. In contrast, those living in LX, LC, HY, JY, and SH increased significantly from Lag 8 to Lag 13 after the typhoon. The greater vulnerability of residents living in the mountainous area (Nanshan) had made the longest duration of mortality risk in LC. Future efforts should focus on accurate identification of vulnerable groups and developing emergency plans for natural disasters. We should adjust measures to local conditions in natural hazards loss, also the contingency plan should be implemented step by step according to current conditions.

### Supplementary Information

**Additional file 1.**

## Data Availability

The daily mortality datasets used and analyzed during the current study are available from the corresponding author on reasonable request. The daily weather data for the same period are collected from the China Meteorological Science Data Sharing Service Network (http://data.cma. cn/). All data generated or analyzed during this study are included in this published article.
