# Supplementary material for: The threaten of typhoons to the health of residents in inland areas: a study on the vulnerability of residents to death risk during typhoon “Lekima”: In Jinan, China
Source: BMC Public Health. 2024 Feb 26;24:606. doi: 10.1186/s12889-024-17667-y (PMC10895747; doi:10.1186/s12889-024-17667-y)
Supplement: Supplementary file 1 — Additional file 1. [file 12889_2024_17667_MOESM1_ESM.docx]

**Supplementary material**

**Additional File 1**


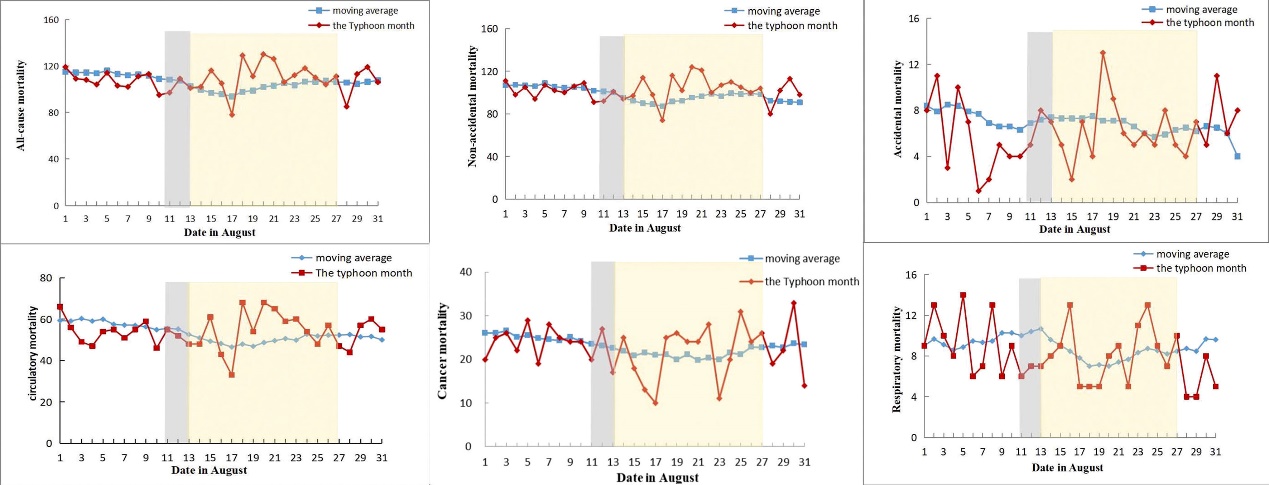


**FigureS1 Daily mortality counts for Jinan residents with different disease in August 2019 compared with average mortality counts in August of previous years**

*The red line: the daily mortality counts for Jinan residents in August 2019;

The blue line: the mean of five-day moving average of mortality in August 2016-2018.

The gray shaded areas: the typhoon period ( August 11 to 13, Aug)

The yellow areas: the period after the typhoon.

**Additional File 2**


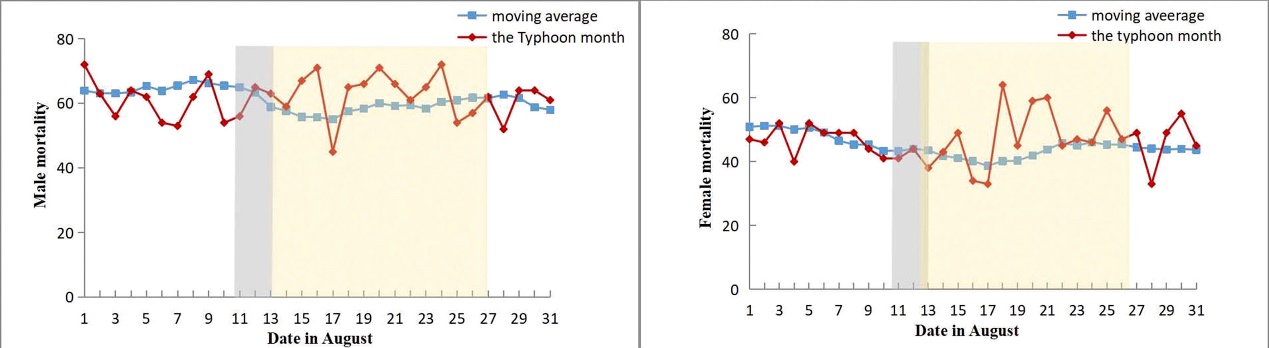


**FigureS2 Daily mortality counts for Jinan residents with different gender in August 2019 compared with average mortality counts in August of previous years**

*The red line: the daily mortality counts for Jinan residents in August 2019;

The blue line: the mean of five-day moving average of mortality in August 2016-2018.

The gray shaded areas: the typhoon period ( August 11 to 13, Aug)

The yellow areas: the period after the typhoon.

**Additional File 3**

**
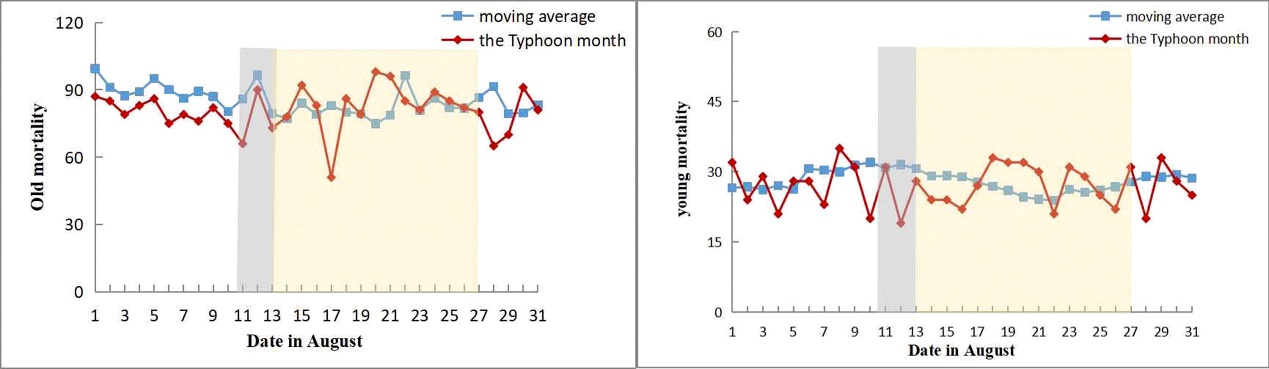
**

**FigureS3** **Daily mortality counts for Jinan residents with different age groups in August 2019 compared with average mortality counts in August of previous years**

*The red line: the daily mortality counts for Jinan residents in August 2019;

The blue line: the mean of five-day moving average of mortality in August 2016-2018.

The gray shaded areas: the typhoon period ( August 11 to 13, Aug)

The yellow areas: the period after the typhoon.

**Additional File 4**


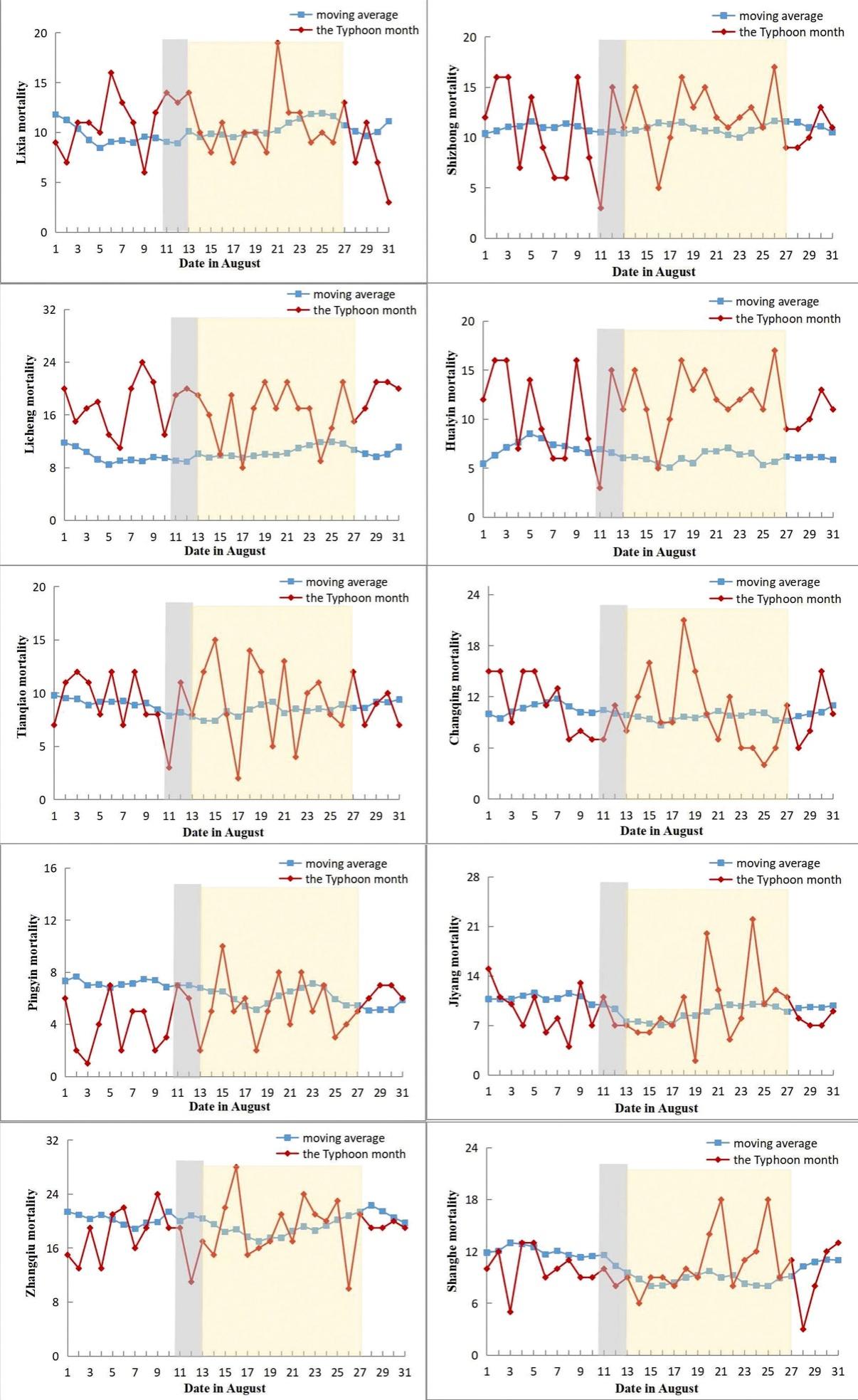


**Figure S4 Daily mortality counts for Jinan residents with different districts in August 2019 compared with average mortality counts in August of previous year**

*The red line: the daily mortality counts for Jinan residents in August 2019;

The blue line: the mean of five-day moving average of mortality in August 2016-2018.

The gray shaded areas: the typhoon period ( August 11 to 13, Aug)

The yellow areas: the period after the typhoon.

**Supplementary Table1**

| **Table S1 Summary single-day lag risk (RR, 95% CI) of death on Jinan residents with different disease during Typhoon** | | | | |
| --- | --- | --- | --- | --- |
| Variable | model | RR | ERL | ERU |
| All-cause | lag0 | 1.069 | 0.921 | 1.240 |
|  | lag1 | 1.058 | 0.920 | 1.215 |
|  | lag2 | 1.059 | 0.929 | 1.208 |
|  | lag3 | 1.081 | 0.951 | 1.230 |
|  | lag4 | 0.992 | 0.868 | 1.134 |
|  | lag5 | 1.010 | 0.887 | 1.151 |
|  | lag6 | 0.989 | 0.869 | 1.127 |
|  | lag7 | **1.129** | **1.002** | **1.272** |
|  | lag8 | **1.144** | **1.017** | **1.287** |
|  | lag9 | **1.168** | **1.039** | **1.313** |
|  | lag10 | **1.131** | **1.001** | **1.278** |
|  | lag11 | 1.112 | 0.983 | 1.259 |
|  | lag12 | 1.124 | 0.995 | 1.271 |
|  | lag13 | 1.122 | 0.989 | 1.274 |
|  | lag14 | 1.048 | 0.920 | 1.193 |
|  | | | | |
| Non-accidental | lag0 | 1.073 | 0.914 | 1.259 |
|  | lag1 | 1.054 | 0.908 | 1.224 |
|  | lag2 | 1.082 | 0.941 | 1.243 |
|  | lag3 | 1.106 | 0.965 | 1.267 |
|  | lag4 | 1.017 | 0.882 | 1.172 |
|  | lag5 | 0.997 | 0.866 | 1.148 |
|  | lag6 | 0.971 | 0.844 | 1.118 |
|  | lag7 | 1.115 | 0.979 | 1.270 |
|  | lag8 | **1.153** | **1.016** | **1.308** |
|  | lag9 | **1.188** | **1.049** | **1.344** |
|  | lag10 | **1.151** | **1.012** | **1.310** |
|  | lag11 | 1.123 | 0.984 | 1.281 |
|  | lag12 | **1.142** | **1.002** | **1.300** |
|  | lag13 | **1.145** | **1.001** | **1.309** |
|  | lag14 | 1.070 | 0.932 | 1.228 |
|  | | | | |
| Accidental | lag0 | 1.012 | 0.587 | 1.743 |
|  | lag1 | 1.112 | 0.663 | 1.863 |
|  | lag2 | 0.731 | 0.414 | 1.291 |
|  | lag3 | 0.728 | 0.415 | 1.278 |
|  | lag4 | 0.645 | 0.364 | 1.143 |
|  | lag5 | 1.207 | 0.780 | 1.869 |
|  | lag6 | 1.257 | 0.822 | 1.922 |
|  | lag7 | 1.331 | 0.880 | 2.015 |
|  | lag8 | 1.009 | 0.621 | 1.639 |
|  | lag9 | 0.881 | 0.525 | 1.479 |
|  | lag10 | 0.827 | 0.487 | 1.404 |
|  | lag11 | 0.964 | 0.591 | 1.573 |
|  | lag12 | 0.885 | 0.536 | 1.459 |
|  | lag13 | 0.821 | 0.488 | 1.381 |
|  | lag14 | 0.743 | 0.438 | 1.259 |
|  | | | | |
| Circulatory | lag0 | 1.148 | 0.930 | 1.417 |
|  | lag1 | 1.037 | 0.847 | 1.270 |
|  | lag2 | 1.053 | 0.872 | 1.271 |
|  | lag3 | 1.008 | 0.834 | 1.218 |
|  | lag4 | 0.897 | 0.738 | 1.091 |
|  | lag5 | 0.948 | 0.782 | 1.148 |
|  | lag6 | 0.967 | 0.802 | 1.165 |
|  | lag7 | 1.174 | 0.992 | 1.388 |
|  | lag8 | **1.187** | **1.004** | **1.404** |
|  | lag9 | **1.255** | **1.069** | **1.475** |
|  | lag10 | **1.213** | **1.027** | **1.434** |
|  | lag11 | 1.142 | 0.960 | 1.360 |
|  | lag12 | 1.095 | 0.913 | 1.312 |
|  | lag13 | 1.096 | 0.909 | 1.321 |
|  | lag14 | 0.997 | 0.825 | 1.207 |
|  |  |  |  |  |
| Respiratory | lag0 | 1.114 | 0.711 | 1.747 |
|  | lag1 | 1.044 | 0.696 | 1.566 |
|  | lag2 | 1.090 | 0.748 | 1.588 |
|  | lag3 | 1.294 | 0.929 | 1.803 |
|  | lag4 | 1.150 | 0.812 | 1.628 |
|  | lag5 | 0.918 | 0.631 | 1.335 |
|  | lag6 | 0.561 | 0.366 | 0.860 |
|  | lag7 | 0.674 | 0.447 | 1.016 |
|  | lag8 | 0.819 | 0.559 | 1.199 |
|  | lag9 | 0.886 | 0.604 | 1.301 |
|  | lag10 | 0.971 | 0.676 | 1.396 |
|  | lag11 | 1.187 | 0.849 | 1.659 |
|  | lag12 | **1.392** | **1.020** | **1.898** |
|  | lag13 | **1.407** | **1.003** | **1.975** |
|  | lag14 | 1.233 | 0.860 | 1.768 |
|  |  |  |  |  |
| Cancer | lag0 | 0.907 | 0.702 | 1.171 |
|  | lag1 | 1.168 | 0.869 | 1.568 |
|  | lag2 | 1.157 | 0.918 | 1.458 |
|  | lag3 | 1.267 | 0.994 | 1.552 |
|  | lag4 | 1.198 | 0.973 | 1.476 |
|  | lag5 | 1.138 | 0.923 | 1.402 |
|  | lag6 | 1.093 | 0.882 | 1.355 |
|  | lag7 | 1.192 | 0.973 | 1.460 |
|  | lag8 | 1.216 | 0.996 | 1.485 |
|  | lag9 | 1.164 | 0.944 | 1.436 |
|  | lag10 | 1.011 | 0.805 | 1.271 |
|  | lag11 | 1.018 | 0.812 | 1.277 |
|  | lag12 | 1.140 | 0.925 | 1.406 |
|  | lag13 | 1.225 | 0.993 | 1.512 |
|  | lag14 | 1.158 | 0.936 | 1.432 |

**Supplementary Table2**

| **Table S2 Summary single-day lag odd risk (RR, 95% CI) of death on Jinan residents with different gender during Typhoon** | | | | |
| --- | --- | --- | --- | --- |
| Variable | model | RR | ERL | ERU |
| male | lag0 | 1.112 | 0.929 | 1.333 |
|  | lag1 | 1.098 | 0.927 | 1.299 |
|  | lag2 | 1.084 | 0.924 | 1.273 |
|  | lag3 | 1.169 | 0.992 | 1.360 |
|  | lag4 | 1.054 | 0.899 | 1.237 |
|  | lag5 | 1.018 | 0.867 | 1.195 |
|  | lag6 | 0.936 | 0.796 | 1.101 |
|  | lag7 | 1.047 | 0.897 | 1.223 |
|  | lag8 | 1.078 | 0.924 | 1.256 |
|  | lag9 | 1.089 | 0.934 | 1.271 |
|  | lag10 | 1.104 | 0.945 | 1.291 |
|  | lag11 | 1.135 | 0.975 | 1.321 |
|  | lag12 | 1.092 | 0.935 | 1.277 |
|  | lag13 | 1.053 | 0.895 | 1.239 |
|  | lag14 | 0.944 | 0.800 | 1.114 |
|  |  |  |  |  |
| female | lag0 | 1.009 | 0.803 | 1.269 |
|  | lag1 | 1.003 | 0.810 | 1.242 |
|  | lag2 | 1.025 | 0.838 | 1.253 |
|  | lag3 | 0.968 | 0.792 | 1.185 |
|  | lag4 | 0.907 | 0.737 | 1.115 |
|  | lag5 | 1.001 | 0.823 | 1.218 |
|  | lag6 | 1.065 | 0.881 | 1.287 |
|  | lag7 | **1.246** | **1.051** | **1.477** |
|  | lag8 | **1.238** | **1.043** | **1.470** |
|  | lag9 | **1.280** | **1.082** | **1.515** |
|  | lag10 | 1.166 | 0.973 | 1.397 |
|  | lag11 | 1.081 | 0.893 | 1.309 |
|  | lag12 | 1.168 | 0.974 | 1.401 |
|  | lag13 | **1.221** | **1.016** | **1.467** |
|  | lag14 | 1.197 | 0.998 | 1.436 |

**Supplementary Table3**

| **Table S3 Summary single-day lag odd risk (RR, 95% CI) of death on Jinan residents with different age groups during Typhoon** | | | | |
| --- | --- | --- | --- | --- |
| Variable | model | RR | ERL | ERU |
| Young(<65) | lag0 | 1.010 | 0.786 | 1.298 |
|  | lag1 | 0.928 | 0.726 | 1.186 |
|  | lag2 | 1.010 | 0.802 | 1.272 |
|  | lag3 | 0.958 | 0.757 | 1.213 |
|  | lag4 | 0.960 | 0.764 | 1.208 |
|  | lag5 | 1.092 | 0.890 | 1.338 |
|  | lag6 | 1.186 | 0.978 | 1.439 |
|  | lag7 | 1.205 | 0.997 | 1.458 |
|  | lag8 | 1.129 | 0.920 | 1.386 |
|  | lag9 | 1.047 | 0.842 | 1.302 |
|  | lag10 | 1.105 | 0.889 | 1.375 |
|  | lag11 | 1.057 | 0.849 | 1.315 |
|  | lag12 | 1.019 | 0.823 | 1.308 |
|  | lag13 | 0.931 | 0.742 | 1.169 |
|  | lag14 | 0.920 | 0.736 | 1.150 |
|  |  |  |  |  |
| old(≥65) | lag0 | 1.090 | 0.914 | 1.301 |
|  | lag1 | 1.003 | 0.810 | 1.242 |
|  | lag2 | 1.025 | 0.838 | 1.253 |
|  | lag3 | 0.968 | 0.792 | 1.185 |
|  | lag4 | 0.907 | 0.737 | 1.115 |
|  | lag5 | 1.001 | 0.823 | 1.218 |
|  | lag6 | 1.065 | 0.881 | 1.287 |
|  | lag7 | 1.246 | 1.051 | 1.477 |
|  | lag8 | 1.238 | 1.043 | 1.470 |
|  | lag9 | 1.280 | 1.082 | 1.515 |
|  | lag10 | 1.166 | 0.973 | 1.397 |
|  | lag11 | 1.081 | 0.893 | 1.309 |
|  | lag12 | 1.168 | 0.974 | 1.297 |
|  | lag13 | 1.221 | 1.016 | 1.467 |
|  | lag14 | 1.197 | 0.998 | 1.436 |
|  |  |  |  |  |
| 65-74 | lag0 | 0.950 | 0.683 | 1.322 |
|  | lag1 | 0.912 | 0.664 | 1.252 |
|  | lag2 | 1.099 | 0.826 | 1.462 |
|  | lag3 | 1.173 | 0.897 | 1.535 |
|  | lag4 | 1.097 | 0.834 | 1.442 |
|  | lag5 | 1.117 | 0.854 | 1.462 |
|  | lag6 | 1.049 | 0.793 | 1.389 |
|  | lag7 | 1.216 | 0.936 | 1.581 |
|  | lag8 | 1.349 | 1.055 | 1.725 |
|  | lag9 | 1.447 | 1.144 | 1.830 |
|  | lag10 | 1.388 | 1.094 | 1.761 |
|  | lag11 | 1.390 | 1.100 | 1.757 |
|  | lag12 | 1.360 | 1.072 | 1.764 |
|  | lag13 | 1.433 | 1.126 | 1.823 |
|  | lag14 | 1.172 | 0.894 | 1.536 |
|  |  |  |  |  |
| 75-84 | lag0 | 1.234 | 0.944 | 1.613 |
|  | lag1 | 1.233 | 0.971 | 1.565 |
|  | lag2 | 1.142 | 0.907 | 1.439 |
|  | lag3 | 1.258 | 1.013 | 1.562 |
|  | lag4 | 1.175 | 0.938 | 1.471 |
|  | lag5 | 1.032 | 0.808 | 1.319 |
|  | lag6 | 0.833 | 0.644 | 1.078 |
|  | lag7 | 1.022 | 0.805 | 1.298 |
|  | lag8 | 1.041 | 0.826 | 1.313 |
|  | lag9 | 1.055 | 0.838 | 1.329 |
|  | lag10 | 1.072 | 0.849 | 1.354 |
|  | lag11 | 1.056 | 0.833 | 1.338 |
|  | lag12 | 1.119 | 0.882 | 1.339 |
|  | lag13 | 0.987 | 0.762 | 1.277 |
|  | lag14 | 0.904 | 0.699 | 1.169 |
|  |  |  |  |  |
| ≥85 | lag0 | 1.064 | 0.779 | 1.454 |
|  | lag1 | 1.141 | 0.861 | 1.513 |
|  | lag2 | 0.953 | 0.715 | 1.270 |
|  | lag3 | 0.871 | 0.651 | 1.166 |
|  | lag4 | 0.648 | 0.473 | 0.889 |
|  | lag5 | 0.751 | 0.555 | 1.018 |
|  | lag6 | 0.895 | 0.677 | 1.184 |
|  | lag7 | 1.091 | 0.843 | 1.414 |
|  | lag8 | 1.104 | 0.852 | 1.430 |
|  | lag9 | 1.200 | 0.932 | 1.545 |
|  | lag10 | 0.970 | 0.735 | 1.281 |
|  | lag11 | 0.951 | 0.715 | 1.264 |
|  | lag12 | 1.006 | 0.761 | 1.256 |
|  | lag13 | 1.227 | 0.949 | 1.586 |
|  | lag14 | 1.291 | 0.943 | 1.646 |

**Supplementary Table 4**

| Table S4 Summary single-day lag odd risk (RR, 95% CI) of death on Jinan residents with different districts during Typhoon | | | | |
| --- | --- | --- | --- | --- |
| Residents | model | RR | ERL | ERU |
| Lixia | lag0 | 1.25 | 0.820 | 1.900 |
|  | lag1 | 0.84 | 0.491 | 1.423 |
|  | lag2 | 0.870 | 0.561 | 1.340 |
|  | lag3 | 0.900 | 0.590 | 1.370 |
|  | lag4 | 0.860 | 0.550 | 1.000 |
|  | lag5 | 0.950 | 0.610 | 1.460 |
|  | lag6 | 0.920 | 0.601 | 1.430 |
|  | lag7 | 0.900 | 0.590 | 1.390 |
|  | lag8 | 1.150 | 0.790 | 1.690 |
|  | lag9 | 1.260 | 0.870 | 1.810 |
|  | lag10 | 1.440 | 1.020 | 2.030 |
|  | lag11 | 1.130 | 0.760 | 1.690 |
|  | lag12 | 1.060 | 0.710 | 1.600 |
|  | lag13 | 0.920 | 0.590 | 1.430 |
|  | lag14 | 1.010 | 0.671 | 1.539 |
|  | | | | |
| Licheng | lag0 | 1.220 | 0.880 | 1.710 |
|  | lag1 | 1.020 | 0.670 | 1.570 |
|  | lag2 | 0.970 | 0.680 | 1.390 |
|  | lag3 | 1.140 | 0.820 | 1.580 |
|  | lag4 | 0.960 | 0.670 | 1.370 |
|  | lag5 | 1.090 | 0.780 | 1.501 |
|  | lag6 | 1.090 | 0.790 | 1.500 |
|  | lag7 | 1.230 | 0.920 | 1.660 |
|  | lag8 | **1.330** | **1.010** | **1.770** |
|  | lag9 | **1.370** | **1.030** | **1.830** |
|  | lag10 | **1.450** | **1.090** | **1.930** |
|  | lag11 | 1.140 | 0.820 | 1.580 |
|  | lag12 | **0.970** | **0.690** | **1.370** |
|  | lag13 | **1.020** | **0.730** | **1.430** |
|  | lag14 | 1.090 | 0.790 | 1.500 |
|  |  |  |  |  |
| Shihzhong | lag0 | 1.070 | 0.670 | 1.720 |
|  | lag1 | 1.710 | 1.060 | 2.750 |
|  | lag2 | 1.180 | 0.790 | 1.780 |
|  | lag3 | 0.950 | 0.630 | 1.430 |
|  | lag4 | 0.800 | 0.520 | 1.250 |
|  | lag5 | 0.950 | 0.630 | 1.430 |
|  | lag6 | 1.180 | 0.810 | 1.710 |
|  | lag7 | 1.310 | 0.920 | 1.870 |
|  | lag8 | 1.140 | 0.790 | 1.660 |
|  | lag9 | 1.130 | 0.770 | 1.640 |
|  | lag10 | 1.030 | 0.700 | 1.520 |
|  | lag11 | 1.120 | 0.760 | 1.640 |
|  | lag12 | 1.140 | 0.770 | 1.670 |
|  | lag13 | 1.480 | 1.020 | 2.140 |
|  | lag14 | 1.250 | 0.850 | 1.850 |
|  |  |  |  |  |
| Huaiyin | lag0 | 1.150 | 0.610 | 2.170 |
|  | lag1 | 1.010 | 0.480 | 2.120 |
|  | lag2 | 1.120 | 0.640 | 1.960 |
|  | lag3 | 0.950 | 0.540 | 1.680 |
|  | lag4 | 1.050 | 0.600 | 1.810 |
|  | lag5 | 1.100 | 0.660 | 1.860 |
|  | lag6 | 1.220 | 0.750 | 1.970 |
|  | lag7 | 1.410 | 0.910 | 2.190 |
|  | lag8 | **1.010** | **0.610** | **1.670** |
|  | lag9 | **1.210** | **0.590** | **1.680** |
|  | lag10 | **0.930** | **0.540** | **1.600** |
|  | lag11 | 1.360 | 0.850 | 2.200 |
|  | lag12 | 1.680 | 1.080 | 2.600 |
|  | lag13 | 1.890 | 1.190 | 3.010 |
|  | lag14 | 1.190 | 0.710 | 2.010 |
|  |  |  |  |  |
| Tianqiao | lag0 | 0.940 | 0.530 | 1.660 |
|  | lag1 | 1.790 | 0.990 | 3.210 |
|  | lag2 | 1.650 | 1.070 | 2.540 |
|  | lag3 | 1.540 | 1.020 | 2.310 |
|  | lag4 | 1.020 | 0.630 | 1.650 |
|  | lag5 | 0.960 | 0.590 | 1.570 |
|  | lag6 | 1.020 | 0.650 | 1.620 |
|  | lag7 | 1.190 | 0.760 | 1.850 |
|  | lag8 | 1.150 | 0.730 | 1.800 |
|  | lag9 | 0.870 | 0.520 | 1.450 |
|  | lag10 | 1.100 | 0.690 | 1.760 |
|  | lag11 | 1.210 | 0.620 | 1.630 |
|  | lag12 | **1.210** | **0.770** | **1.890** |
|  | lag13 | **1.010** | **0.620** | **1.660** |
|  | lag14 | 1.100 | 0.680 | 1.790 |
|  |  |  |  |  |
| Changqing | lag0 | 1.010 | 0.600 | 1.700 |
|  | lag1 | 1.400 | 0.750 | 2.610 |
|  | lag2 | 1.380 | 0.990 | 2.120 |
|  | lag3 | 1.450 | 0.980 | 2.150 |
|  | lag4 | 1.260 | 0.830 | 1.890 |
|  | lag5 | 1.550 | 1.070 | 2.240 |
|  | lag6 | 1.580 | 1.120 | 2.230 |
|  | lag7 | 1.410 | 1.112 | 2.010 |
|  | lag8 | 0.960 | 0.630 | 1.470 |
|  | lag9 | 0.930 | 0.600 | 1.440 |
|  | lag10 | 0.870 | 0.540 | 1.400 |
|  | lag11 | 0.820 | 0.510 | 1.320 |
|  | lag12 | 0.580 | 0.330 | 1.020 |
|  | lag13 | 0.570 | 0.320 | 1.010 |
|  | lag14 | 0.670 | 0.410 | 1.110 |
|  |  |  |  |  |
| Pingyin | lag0 | 1.080 | 0.660 | 1.750 |
|  | lag1 | 0.590 | 0.320 | 1.080 |
|  | lag2 | 0.880 | 0.570 | 1.350 |
|  | lag3 | 1.100 | 0.750 | 1.610 |
|  | lag4 | 1.110 | 0.770 | 1.610 |
|  | lag5 | 0.750 | 0.470 | 1.180 |
|  | lag6 | 0.680 | 0.430 | 1.070 |
|  | lag7 | 0.750 | 0.490 | 1.150 |
|  | lag8 | 0.820 | 0.550 | 1.240 |
|  | lag9 | 0.980 | 0.670 | 1.440 |
|  | lag10 | 0.860 | 0.570 | 1.290 |
|  | lag11 | 1.020 | 0.700 | 1.490 |
|  | lag12 | 0.880 | 0.570 | 1.360 |
|  | lag13 | 0.870 | 0.550 | 1.380 |
|  | lag14 | 0.690 | 0.420 | 1.120 |
|  |  |  |  |  |
| Jiyang | lag0 | 1.120 | 0.610 | 2.070 |
|  | lag1 | 0.710 | 0.320 | 1.570 |
|  | lag2 | 0.710 | 0.370 | 1.360 |
|  | lag3 | 0.820 | 0.450 | 1.500 |
|  | lag4 | 0.770 | 0.430 | 1.380 |
|  | lag5 | 0.900 | 0.530 | 1.530 |
|  | lag6 | 0.630 | 0.350 | 1.140 |
|  | lag7 | 1.110 | 0.680 | 1.810 |
|  | lag8 | 1.290 | 0.810 | 2.060 |
|  | lag9 | 1.480 | 0.940 | 2.320 |
|  | lag10 | 0.990 | 0.570 | 1.710 |
|  | lag11 | 1.320 | 0.830 | 2.090 |
|  | lag12 | 1.530 | 0.990 | 2.350 |
|  | lag13 | 1.920 | 1.260 | 2.900 |
|  | lag14 | 1.340 | 0.810 | 2.220 |
|  |  |  |  |  |
| Zhangqiu | lag0 | 0.870 | 0.600 | 1.260 |
|  | lag1 | 0.880 | 0.560 | 1.370 |
|  | lag2 | 1.040 | 0.740 | 1.450 |
|  | lag3 | 1.150 | 0.850 | 1.540 |
|  | lag4 | 1.130 | 0.840 | 1.510 |
|  | lag5 | 0.970 | 0.710 | 1.310 |
|  | lag6 | 0.820 | 0.590 | 1.150 |
|  | lag7 | 0.960 | 0.700 | 1.330 |
|  | lag8 | 1.070 | 0.780 | 1.480 |
|  | lag9 | 1.140 | 0.850 | 1.550 |
|  | lag10 | 1.080 | 0.800 | 1.450 |
|  | lag11 | 1.120 | 0.840 | 1.500 |
|  | lag12 | 1.060 | 0.790 | 1.420 |
|  | lag13 | 0.930 | 0.670 | 1.300 |
|  | lag14 | 0.980 | 0.710 | 1.360 |
|  |  |  |  |  |
| Shanghe | lag0 | 0.980 | 0.610 | 1.610 |
|  | lag1 | 0.720 | 0.410 | 1.320 |
|  | lag2 | 0.820 | 0.510 | 1.340 |
|  | lag3 | 0.850 | 0.530 | 1.360 |
|  | lag4 | 0.970 | 0.620 | 1.530 |
|  | lag5 | 0.930 | 0.610 | 1.440 |
|  | lag6 | 0.870 | 0.560 | 1.340 |
|  | lag7 | 1.050 | 0.710 | 1.580 |
|  | lag8 | 1.410 | 0.980 | 2.030 |
|  | lag9 | 1.430 | 0.990 | 2.060 |
|  | lag10 | 1.360 | 0.930 | 1.990 |
|  | lag11 | 1.170 | 0.770 | 1.770 |
|  | lag12 | 1.520 | 1.070 | 2.170 |
|  | lag13 | 1.480 | 1.100 | 2.170 |
|  | lag14 | 1.370 | 0.930 | 2.030 |
